# Supplementary figures and images for: Pupillary dynamics of mice performing a Pavlovian delay conditioning task reflect reward-predictive signals
Source: Front Syst Neurosci. 2022 Dec 8;16:1045764. doi: 10.3389/fnsys.2022.1045764 (PMC9772849; doi:10.3389/fnsys.2022.1045764)

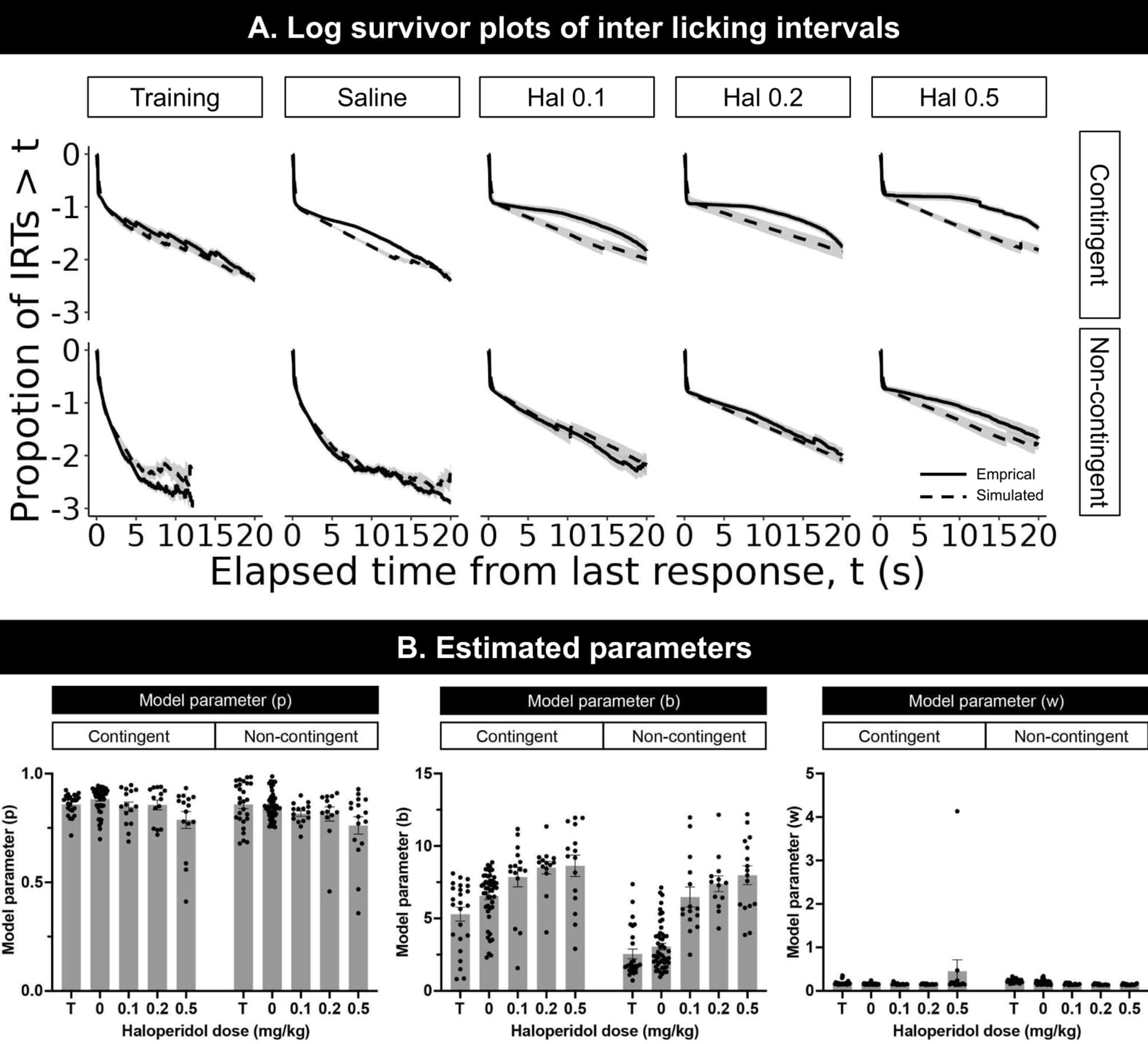

Supplement: Supplementary Figure 1 — Empirical contingency in the non-contingent group. We calculated the percentage of CS and US overlapping trials for all individuals and sessions in the non-contingent group. The definition of overlap was determined by whether reward presentations were presented during auditory stimulus presentations. The log survivor plot is a method to visualize the bout-and-pause patterns, and when responses have bout-and-pause patterns, the plot shows the broken-stick curve. The left side line denotes the within-bout inter-licking intervals, and the right-side line denotes the bout initiation intervals. The intercept of the right-side line denotes the bout length, the amount of licking contained in one bout. Log survivor plots of empirical and simulated data showed broken-stick curves, suggesting licks have bout-and-pause patterns. As shown in the figure, the bend points were ~0.1–1.0 s, suggesting that the boundaries separating the within-bout licking from the bout initiation licking were in the range and corresponded to Figures 3B,C. We found that the log survivor plot in the contingent group showed a clear bend point in training and saline conditions; in contrast, the plot did not show a clear bend point and showed a gradual curve in the non-contingent group. As the dose of haloperidol increased, the bend point became clearer. In the contingent group, the slope of the right lines became gradual as the dose of haloperidol increased. Taken together, mice did not show spontaneous licking during the inter-reward intervals. [file Image_1.JPEG]

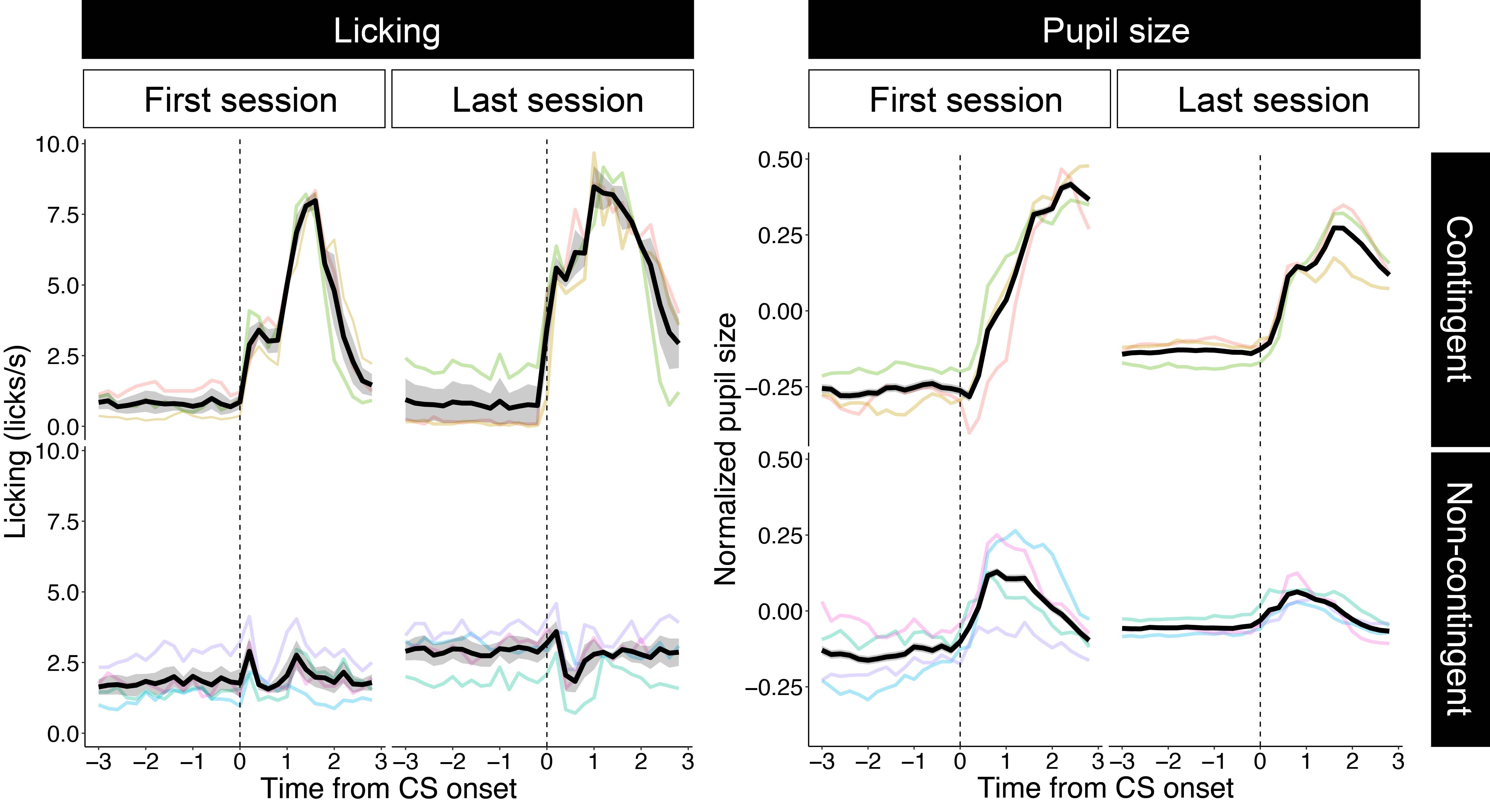

Supplement: Supplementary Figure 2 — Fitting results of the mixture exponential distribution. (A) Solid lines show log survivor plots of empirical inter-licking intervals, and dashed lines show log survivor plots of simulated data using fitted parameters. Model fitting was performed independently for individual and session data, and we generated random numbers from the distribution of the estimated parameters. Each line denotes the average over subjects and sessions, and gray shades denote standard errors. (B) Average value and range of estimated parameters, w, b, and p, in each group and dose condition. T denotes the data in the last three sessions of training. We compared the dynamics of licking and pupil size before/after the auditory stimulus presentation between the first and last training sessions. However, we failed to record several data in the first session, showing only four and three subjects for contingent and non-contingent groups. In the first session, the amount of licking slightly increased after the auditory stimulus in the contingent group but not in the non-contingent group. In the last session, the increase in the amount of licking became larger in the contingent group. In the non-contingent group, the amount of licking did not increase after the auditory stimulus presentation, but the baseline was larger in the last session compared to the first session. In both groups, mice showed pupil dilation to the auditory stimulus in the first session, but in the last session, it decreased in the non-contingent group but not in the contingent group. The amount of licking was acquired by Pavlovian conditioning, but the pupil size showed an increase in the very first session. The pupil size is highly correlated with LC activity, and LC shows the phasic activity to a novel stimulus. It also shows the activity when the environmental rule, such as stimulus-reward contingency, changes. The increase in pupil size in the first session may reflect the novelty of stimulus or change in the environmenta [file Image_2.JPEG]

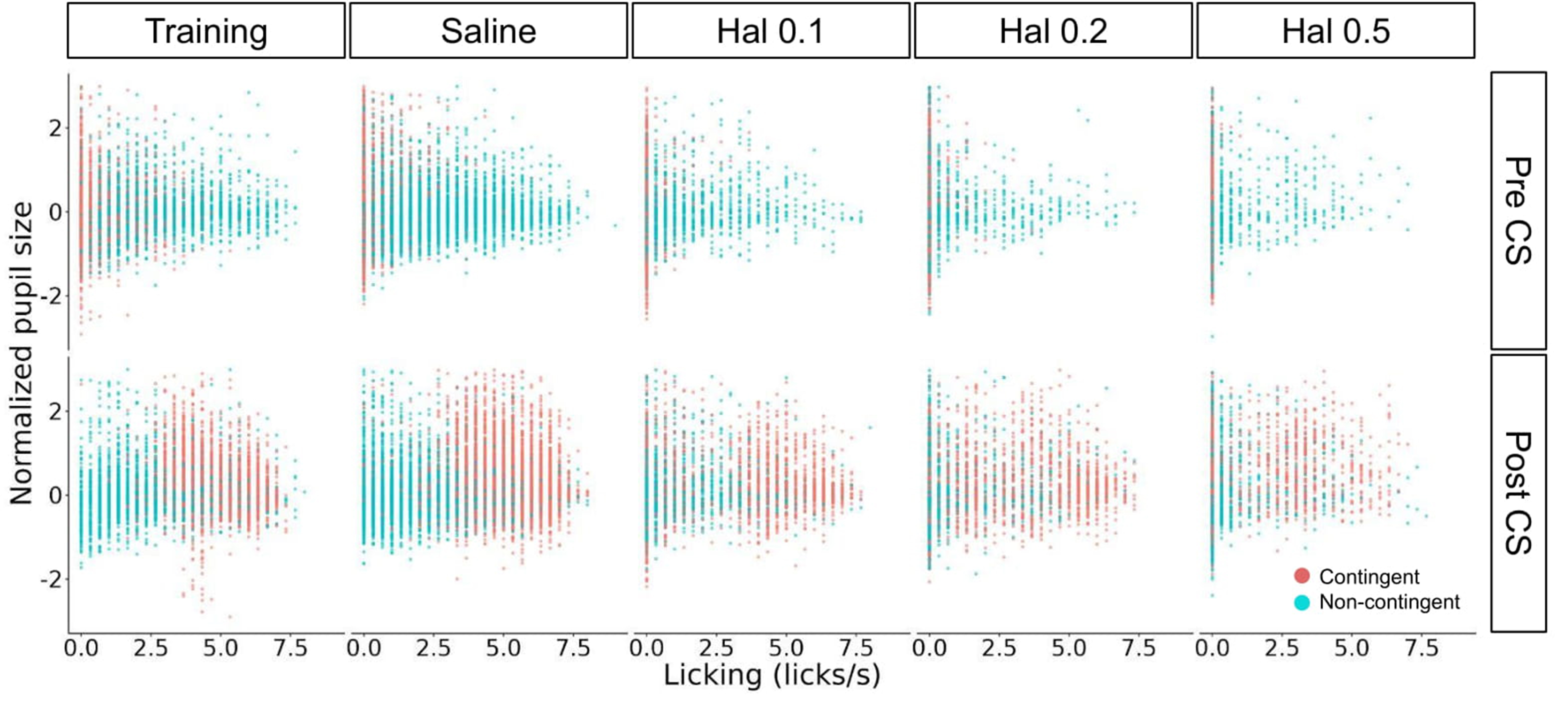

Supplement: Supplementary Figure 3 — Comparison of dynamics of licking and pupil size between early and last session in training. We analyzed the relationship between the amount of licking and pupil size in each trial. Although licking increased pupil size, as shown in Figure 3, we could not find any relationship between the amount of licking and pupil size. The large temporal variance in pupil size may mask the relationship between licking and pupil size in this time-scale analysis. [file Image_3.JPEG]

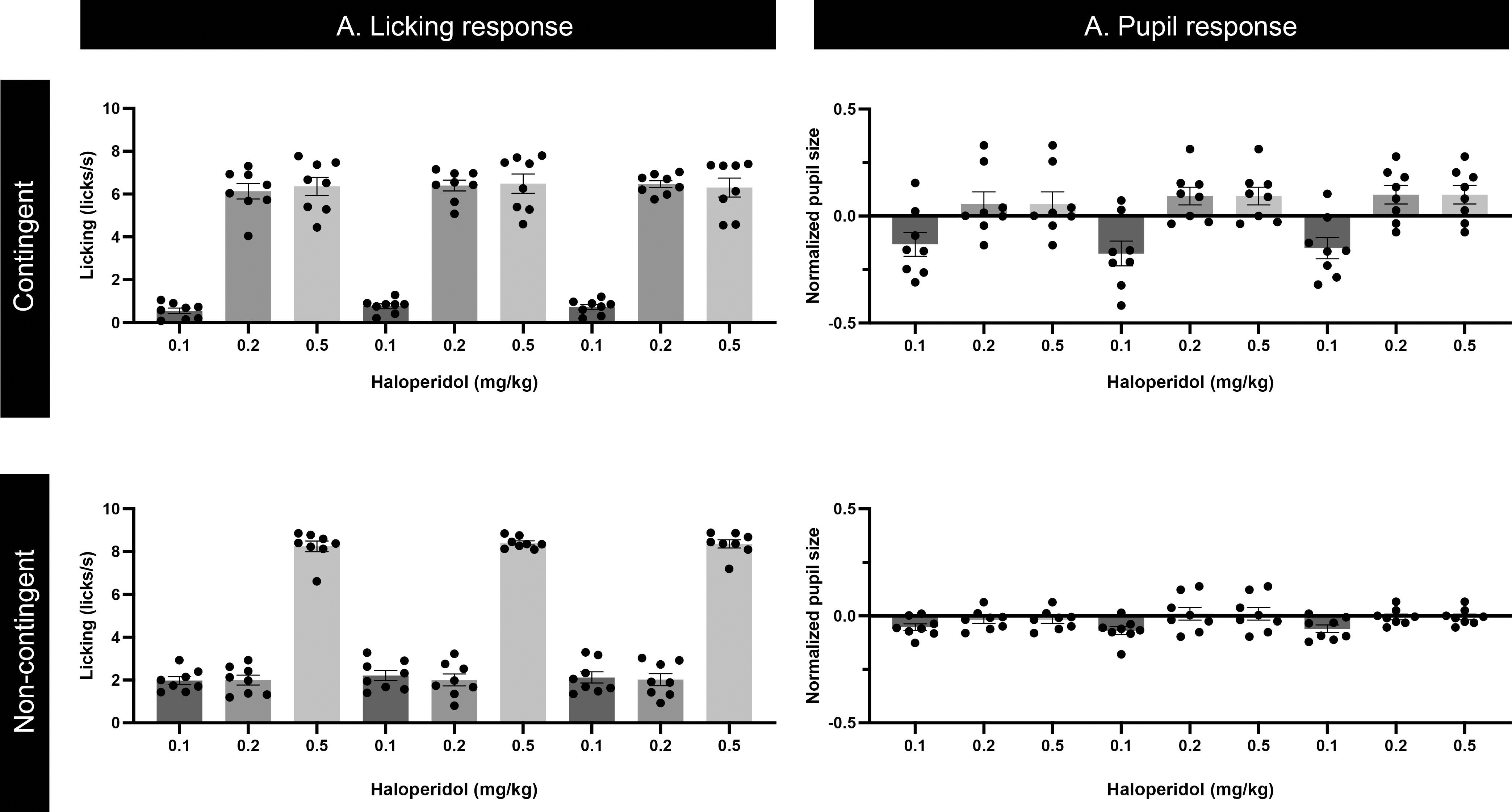

Supplement: Supplementary Figure 4 — Scatter plots of the amount of licking and pupil size. The upper panels show relationships between the amount of licking and pupil size at 3 s before the auditory stimulus presentation. The bottom panels show those of 3 s after the auditory stimulus presentation. Blue and red points denote the non-contingent and contingent groups, respectively. We analyzed the saline condition separately for previous haloperidol dose conditions to examine whether the effects of haloperidol were washed-out. We found no difference between the previous dose in the amount of licking and pupil size at any time window, suggesting that the effects of haloperidol had been washed-out until the saline condition. [file Image_4.JPEG]

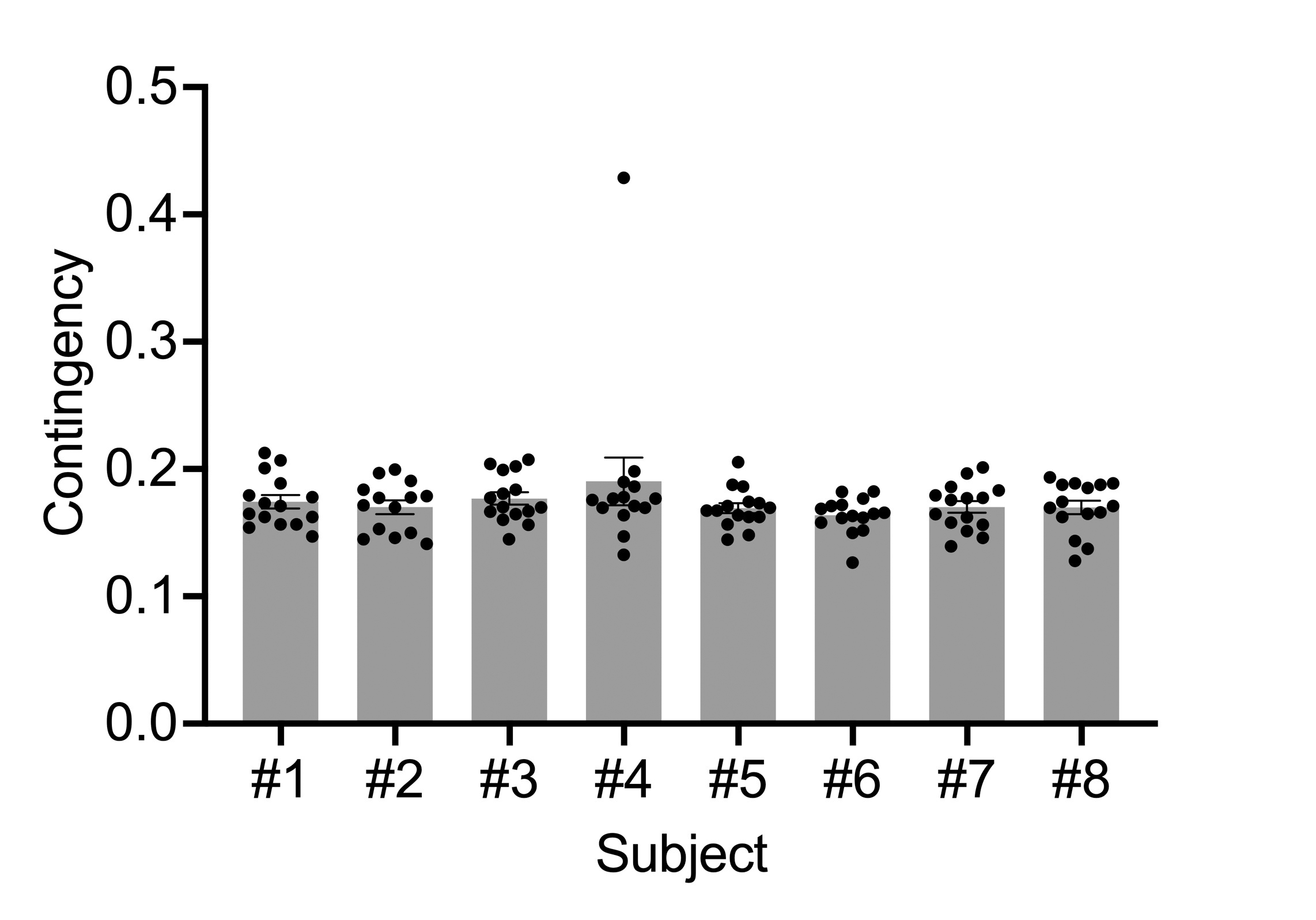

Supplement: Supplementary Figure 5 — The amount of licking and pupil size at the time windows. The amount of licking and pupil size in Pre-CS, CS, and US periods of saline condition were separately shown by the previous dose of haloperidol injection. [file Image_5.JPEG]
